# Change in radial artery pulse wave in stroke hemiplegic patients: Protocol for a case-control study

**DOI:** 10.1097/MD.0000000000010204

**Published:** 2018-03-30

**Authors:** Jaeuk U. Kim, Jae Kyoun Kim, Jae-Young Shin, Boncho Ku, Jang-Han Bae, Seungryong Yeom, Sangkwan Lee

**Affiliations:** a KM Fundamental Research Division, Korea Institute of Oriental Medicine, Daejeon; b Clinical Trial Center, Wonkwang University Gwangju Hospital, Gwangju; c Department of Global Public Health and Korean Medicine Management, Graduate School, Kyung Hee University, Seoul; d Clinical Research Division, Korea Institute of Oriental Medicine, Daejeon; e College of Korean Medicine, Wonkwang University, Iksan, Jeonbuk, Republic of Korea.

**Keywords:** hemiplegia, matched case-control study, protocol, radial artery pressure pulse wave, stroke

## Abstract

**Introduction:**

About 55% to 75% of stroke survivors have motor disorders and problems that affect their quality of life. The prevention of secondary neurological damages through relapse prevention and the rehabilitation of stroke patients suffering from morbidities are crucial to improve the prognosis of patients with stroke. Pulse examinations can be used to determine the stroke progression. This study will investigate the differences and changes in radial artery pressure-pulse waves during the treatment of hemiplegia caused by stroke.

**Methods/design:**

This study protocol is for a prospective matched case-control study. A total of 84 participants will be recruited, 56 patients with hemiplia caused by stroke, and 28 control patients matched by age, gender, and body mass index. The primary outcome of this study will be the differences and changes in the radial augmentation index.

**Discussion:**

The results of the study will help to determine the differences and changes in radial artery pressure-pulse waves during the treatment of hemiplegia caused by stroke. The findings will provide information about the physiological and hemodynamic mechanisms.

**Conclusion:**

This will be the first study to analyze the pulse wave of the radial artery (PWRA) on the affected side and on the normal side in stroke patients with hemiplegia. This study will clarify whether the radial artery pressure pulse wave can be used to evaluate the result of stroke treatment objectively. The results of the study will be available in February 2019. The version of the protocol is v1.6 written in March 7, 2016.

**Ethics and dissemination:**

Written informed consent will be obtained from all participants. This study has been approved by the Institutional Review Board (IRB) of Wonkwang University Gwangju Hospital, Gwangju, Republic of Korea (WKIRB-2016/8). The study findings will be published in peer-reviewed journals and presented at national and international conferences.

**Trial registration number:**

This trial was registered with the Clinical Research Information Service (CRIS) of the Korea National Institute of Health (NIH), Republic of Korea (KCT0002147).

## 1 Background

About 55% to 75% of stroke survivors have motor disorders and problems that affect their quality of life.^[1]^ These patients often show motor disorders associated with hemiplegia that limit their physical activities. Stroke has a high rate of relapse and leads to serious morbidities, including motor disorders, gait abnormality, and speech disorders. Therefore, the prevention of secondary neurological damages through relapse prevention and the rehabilitation of stroke patients suffering from morbidities are crucial to improve the prognosis of patients with acute ischemic stroke.^[2]^

Accurate stroke diagnoses can be made with computed tomography and magnetic resonance imaging. However, the clinical applications of these medical imaging techniques are limited, as it is difficult to install the equipment and to obtain repeated measurements to monitor the progress of the treatment, and expensive examination fees are involved.^[3]^

The risk of stroke is proportional to the arterial stiffness. In the case of atherosclerosis, the arteries have reduced elasticity and increased arterial stiffness, arterial pulse wave velocity, and pulse pressure. The pulse wave velocity (PWV) is an independent predictor of cardiovascular disease mortality, and an important predictor of the risk of atherosclerosis. It is a relatively simple method to assess the changes in arteries, and has the advantage of being repeatable and noninvasive. PWV measurements can predict the onset of stroke or cardiovascular disease, which can help to select a more appropriate treatment.

Pulse examinations are closely associated with motor function improvement through rehabilitation and exercise therapy, and with changes in the blood flow.^[4–7]^ These blood flow changes can affect the pulse waves in the radial arteries. Therefore, pulse examinations can be used to determine the stroke progression.

The arterial pulse can be measured noninvasively in superficial arteries-such as the carotid, brachial, radial, and femoral arteries-using applanation tonometry. In Eastern medicine, doctors analyze not only the temporal, but also the spatial characteristics of the pulse waves in the left and right radial arteries to diagnose diseases and to assess the effectiveness of treatments.

We will compare the pulse waves and hemodynamic characteristics of the affected and non-affected radial artery pulses in patients with stroke hemiplegia, and will assess the correlation between the brachial-ankle PWV (baPWV) and the blood viscosity to evaluate the stroke prognosis.

The aim of this study is to measure the pulse wave of the radial artery in stroke patients with hemiplegia, and to compare the changes in the pulse waves of the radial arteries on the affected and less affected sides of hemiplegic patients after rehabilitation by physiotheraphy, as well as to evaluate the effects of increased physical activities on the pulse wave of the radial artery (PWRA).

## 2 Methods

### 2.1 Study design

This study was conceived as a prospective and observational study to explore the differences and changes in radial artery pulse waves during the treatment of patients over 50 years old suffering from hemiplegia caused by stroke. As a matched case-control study, it aims to compare the pulse waves in the radial arteries of participants with hemiplegia caused by stroke with those of healthy participants. The study will be carried out at the Wonkwang University Gwangju Hospital Clinical Trial Center of Gwangju, in the Republic of Korea.

### 2.2 Study duration

The study period is from August 1, 2016 to December 15, 2018.

### 2.3 Ethics statement and trial registration

The study protocol was approved by the Institutional Review Board of Wonkwang University Gwangju Hospital in Gwangju, Republic of Korea (WKIRB-2016/8), and is registered with the Clinical Research Information Service (CRIS) of the Korea National Institute of Health (NIH) in the Republic of Korea (KCT0002147), which is a WHO Registry Network registry. Written informed consent will be obtained from all participants, in accordance with the Declaration of Helsinki.

### 2.4 Participants

A total of 84 participants will be enrolled in this matched case-control study. The cohort will comprise 56 patients with hemiplegia caused by stroke, and half the number of control patients matched by age, gender, and body mass index (BMI). The 56 participants with hemiplegia caused by stroke and the 28 healthy participants will be recruited through advertisements, and the 56 patients with stroke hemiplegia will be recruited among the outpatients or inpatients of the Wonkwang University Gwangju Hospital. Each participant will receive an explanation of the study procedure and will be asked to sign the consent form. The patients’ vital signs and relevant histories will be recorded.

#### 2.4.1 Inclusion criteria

- Case group

(1) Men and women over 50 years old.
(2) Patients with stroke diagnosed by computed tomography (CT) or magnetic resonance imaging (MRI) scans for the first time.
(3) Patients with a stroke experience within the past 6 months.
(4) Patients who have required hospital treatment to treat an ischemic stroke.
(5) Patients with a manual muscle testing (MMT) score of 4 or less.
(6) Patients with scores of 24 or more in the Mini Mental State Examination-Korean version (MMSE-K) and with no cognitive functional disability.
(7) Patients who understand and are willing to comply with all of the test procedures, and who participate in the clinical study voluntarily.
(8) Patients who have signed and submitted a consent form for participation in the clinical study.

-Control group

(1) Men and women over 50 years old.
(2) Subjects who have not been diagnosed with strokes.
(3) Subjects with an MMT score of 5.
(4) Subjects with an MMSE-K score of 24 points or higher and no cognitive functional disability.
(5) Subjects who understand and are willing to comply with all of the test procedures, and who participate in the clinical study voluntarily.
(6) Subjects who have signed and submitted a consent form for participation in the clinical study.

#### 2.4.2 Exclusion criteria

- Case group

(1) Patients diagnosed with a hemorrhagic stroke through CT or MRI scans.
(2) Patients whose pulse waves cannot be easily measured due to malformation of their blood vessels around the wrist or to bone fractures, or patients who cannot comply with the test procedure for other reasons.
(3) Patients with serious organic diseases (cardiac disorders or lung, liver, or kidney diseases), or a history of a central nervous system or psychiatric disorder or of taking psychoactive drugs.
(4) Patients with neurological disorders other than strokes.
(5) Patients with a SBP < 90 mmHg or > 220 mmHg, and/or a DBP < 40 mmHg or > 130 mmHg.
(6) Patients with severe musculoskeletal diseases from injury or rheumatic diseases.
(7) Pregnant or nursing women.
(8) Patients with implanted cardiac pacemakers and electronic devices.
(9) Patients who show skin trouble around the electrode attachment region or side effects during the electrode attachment.
(10) Patients who cannot comply with the stroke clinical pathway (CP) provided by the treatment institution.
(11) Patients with an unstable heartbeat.
(12) Patients with a history of drug or alcohol abuse.
(13) Patients who have participated in other clinical trials in the past 3 months.
(14) Patients who have an abnormal reaction to traditional Korean medicine or acupuncture.
(15) Patients considered inappropriate for the clinical study by the research head or staff.

- Control group

(1) Subjects whose pulse waves cannot be easily measured due to malformation of their blood vessels around the wrist or to bone fractures, or subjects who cannot comply with the test procedure for other reasons.
(2) Subjects with serious organic diseases (cardiac disorders or lung, liver, or kidney diseases), or a history of a central nervous system or psychiatric disorder or of taking psychoactive drugs.
(3) Subjects with severe musculoskeletal diseases from injury or rheumatic diseases.
(4) Subjects with a SBP < 90 mmHg or > 220 mmHg, and/or a DBP < 40 mmHg or > 130 mmHg.
(5) Pregnant or nursing women.
(6) Subjects with implanted cardiac pacemakers or electronic devices.
(7) Subjects who show skin trouble around the electrode attachment region or side effects during the electrode attachment.
(8) Subjects with an unstable heartbeat.
(9) Subjects with a history of drug or alcohol abuse.
(10) Subjects who have participated in other clinical tests in the past 3 months.
(11) Subjects considered inappropriate for the clinical study by the research head or staff.

#### 2.4.3 Participant recruitment

The participants will be recruited through advertisements on hospital websites and on bulletin boards. They will be fully briefed on the study, including on the measuring methods and the procedures used. The subjects interested in participating will be guided through the informed consent process by the investigator on their first visit. Once their written consent is obtained, they will be screened using inclusion/exclusion criteria to determine their eligibility. If eligible, a physician will acquire the baseline data, and the clinical research coordinator will then schedule the study procedure. The participant recruitment will be stratified by age, gender, and BMI.

#### 2.4.4 Sample size

The sample size calculation was based on the 2010 age (50 years or older) and gender distribution data provided by the Korean Statistical Information Service (kosis.kr). The incidence rates of obesity and stroke were estimated according to the National Nutrition Survey conducted and released in 2014. The stratification variables were as follows: age (A_1_: 50–69 years; A_2_: 70 years or older), gender (S_1_: male; S_2_: female), and obesity status (O_1_: BMI < 25 kg/m^2^, O_2_: BMI ≥ 25 kg/m^2^). A total of 8 cells (strata) were created based on combinations of the 3 variables. The frequencies of the stratification variables in each cell are shown in the table.

The Neyman allocation method was used, and the sample size was calculated with a confidence interval of 95% (α=0.05) and a margin of error of Є=10% following equation was used to calculate the total sample size and the sample size for each stratum:^[8]^ 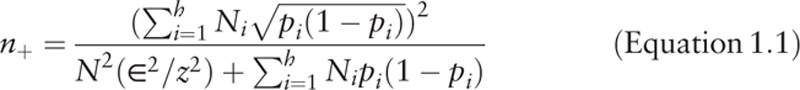 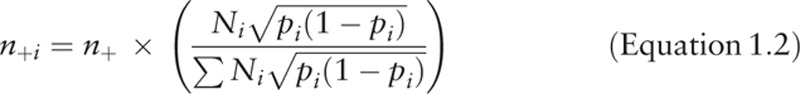


*N* refers to the total number of stroke patients in the corresponding age group, *N*_*i*_ to the size of the *i* stratum of the patient group, *n*_+_ to the sample size of the patient group, *p*_+_ to the population ratio of the *i* stratum, z_α/2_ to the top 100 (α/2)% quantile in a standard normal distribution, Є to the margin of error, and *n*_+*i*_ to the sample size of the *i* stratum, where *i* = 1, …,8 and *h* = 8. The standard deviation 
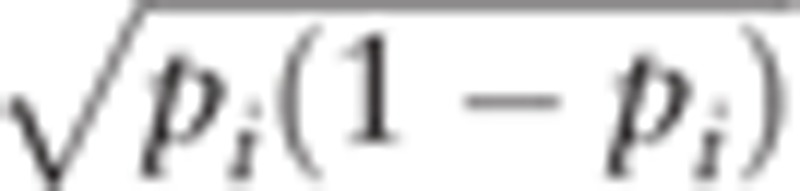
 of the population in each stratum was derived from the information.

The sample size of the patient group was calculated according to the level of significance and the margin of previously selected errors as *n*_+_ = 47. The final target sample size for the stroke patient group was 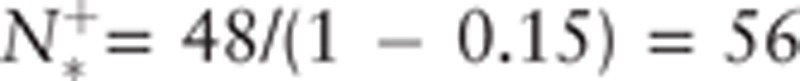, accounting for a 15% drop-out rate. (Table 1)

**Table 1:**
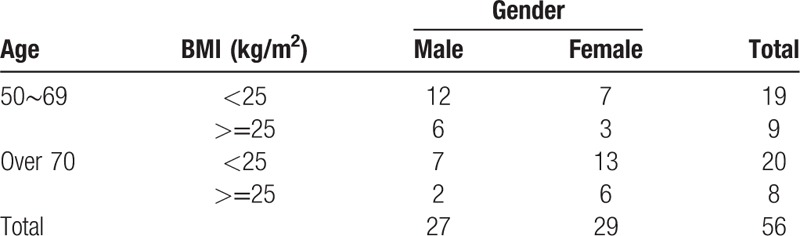
Sample allocation (case group).

A total of 28 patients will be recruited into the control group (0.5 time the sample size of the patient group) for comparison. The proportionate allocation results of the 28 control patients will be selected for frequency matching for each stratum, using the same ratio as in the stroke patient group. (Table 2)

**Table 2:**
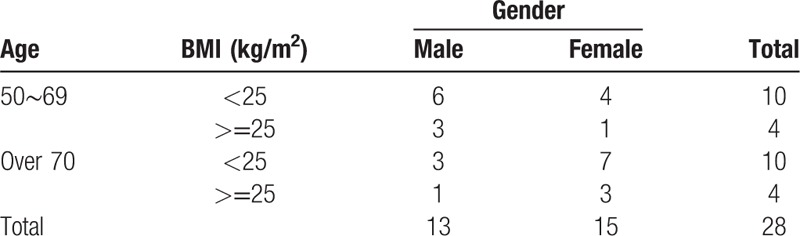
Sample allocation (control group).

#### 2.4.5 Randomization and blinding

This clinical study was designed as a case-control study. Therefore, consideration of the method of randomization and blinding is not needed.

#### 2.4.6 Collection of demographic and lifestyle information

The medical history of the participants, including their current medication status and surgical history, the presence of other diseases, and the results of their electrocardiography and pregnancy tests, will be recorded at baseline. Lifestyle factors including exercise, smoking, caffeine intake, and alcohol consumption will also be documented. In addition, the patients’ hypertension, height, weight, and other demographic information will be obtained.

### 2.5 Data collection

The data will be collected through a participant survey and through investigator measurements at baseline (on visit 1) and at specified follow-up times (on visits 2 to 7). (Table 3)

**Table 3:**
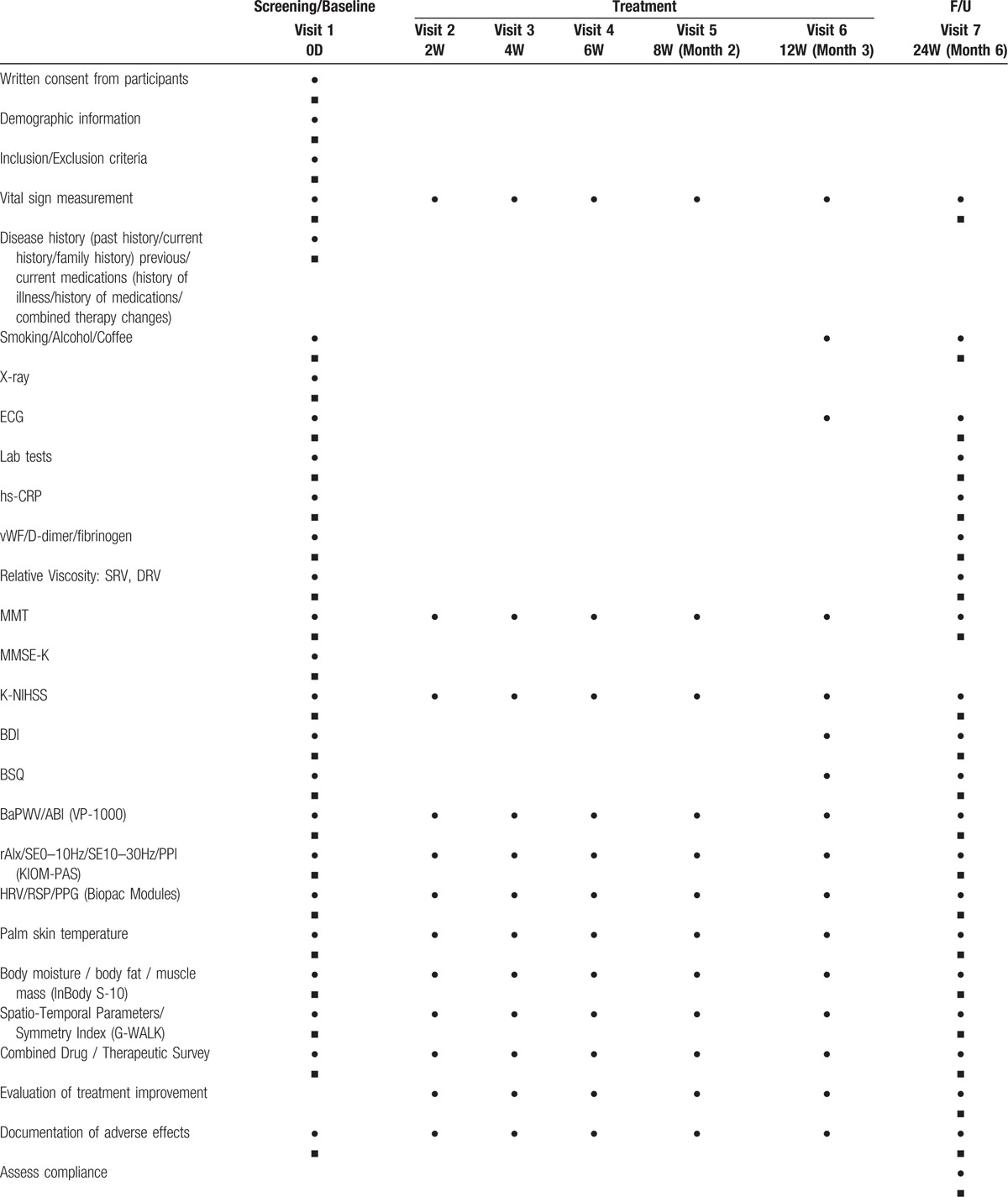
Schedule for study procedure.

### 2.6 Devices and measurement techniques

#### 2.6.1 Pulse tonometric device (KIOM-PAS)

The KIOM-PAS will be acquired with a pulse tonometric device developed at the Korean Institute of Oriental Medicine in Daejeon, Korea. This device consists of a main body with an arm holder and a sensing body attached to a mobile actuator. A pulse detection sensor composed of 7 piezoresistive unit sensors within 9×9 mm^2^ is located at the actuator tip. A previous study we conducted found that the radial artery was more superficial at Gwan (Guan: prominent bone) than Chon (Cun) and Cheok (Chi).^[9]^ Therefore, in this study, the operator will measure the pulse signals at Gwan.

#### 2.6.2 Physiological data acquisition system (Biopac module)

The physiological data will be obtained with a Biopac MP150 (Biopac module, Biopac Systems Inc., USA). The heart rate (HRV) signal will be obtained with an ECG100C. The respiration (RSP) signal will be obtained with a SKT100C. The mean AUC of the photoplethysmogram (MAUGppg) signal will be obtained with a PPG100C.

#### 2.6.3 Blood viscosity measurement (VISCORE-300)

Viscore-300 is a device designed to measure the viscosity with a V-Chip. This chip enables measurement from a small quantity of fluid (0.5cc) and consists of a lab-on-a-chip, a syringe, and a mounting case. The lab-on-a-chip is fitted with 150 microfluidic channels (50 μm), allowing the viscosity to be measured with high precision and replication. The viscosity will be determined by measuring the relative viscosity between the reference fluid and the sample fluid.

### 2.7 Outcome measures

#### 2.7.1 Primary outcome

**Figure 1:**
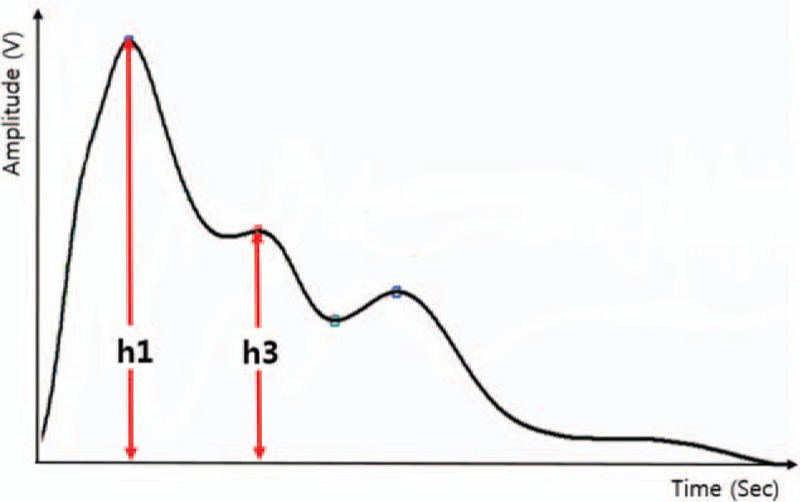
Single pulse waveform at radial artery as a function of time.

Radial augmentation index (rAIx) The radial augmentation index (rAIx) measures the vascular compliance and stiffness of the radial arterial vessel walls, that is, the degree of variation in the magnitude of the pulse wave by the reflected wave. The radial augmentation will be calculated from the radial wave pulse as follows: (second peak SBP (SBP2) DBP)/(first peak SBP_DBP)_100,13, as shown in Figure 1.

#### 2.7.2 Secondary outcomes

(1) Spectrum energy 0–10 Hz (SE0–10 Hz)/Spectrum energy 10–30 Hz (SE10–30 Hz)

The power spectral density of 60-second pulse waves will be obtained under constant hold-down pressure. The width values of the power spectral densities within 0–10 Hz and 10–30 Hz variations will be calculated.

(2) Pulse power index (PPI)

**Figure 2:**
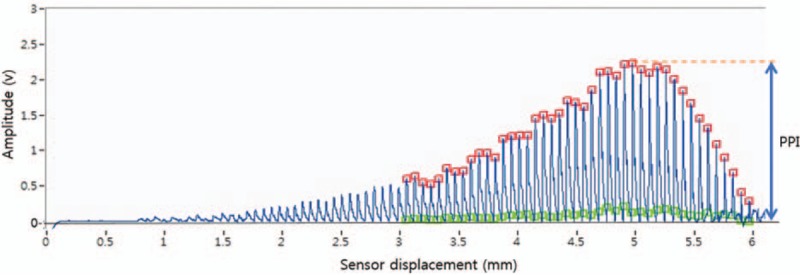
Pulse waveform extraction as a function of sensor displacement from the skin contact point of the measurement sensor.

The pulse waveform initiation point and peak of the pulse waves will be extracted, and the largest peak amplitude (at optimal hold-down pressure) will be defined as the pulse power index, as shown in Figure 2.

(3) Heart rate variability (HRV)

**Figure 3:**
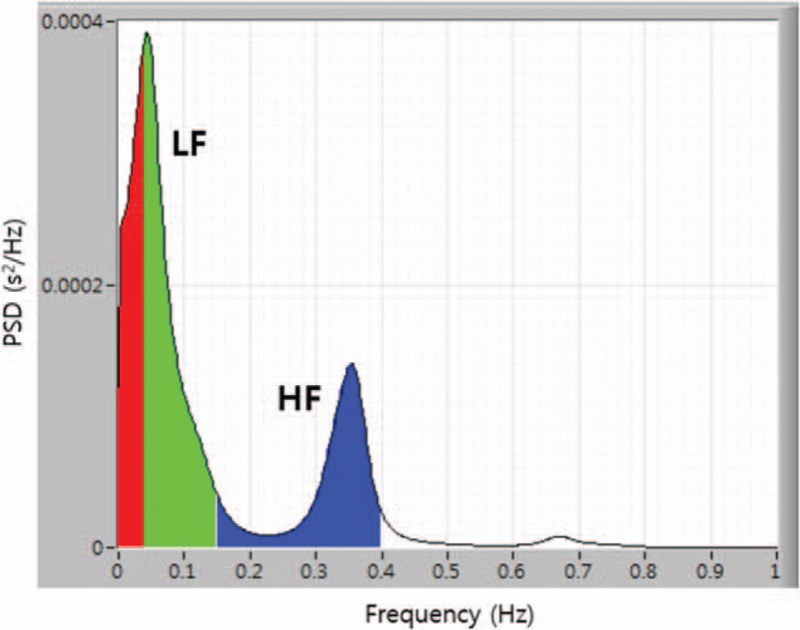
Power spectral density (PSD) of HRV signal obtained from electrocardiogram.

The heart beat intervals between the R peaks of the electrocardiograms will be Fourier-transformed to obtain the frequency domain power spectrum of the heart rate variability. LF (0∼0.15 Hz) area mainly reflects the activity of the sympathetic nervous system and HF (0.15∼0.4 Hz) area reflects the activity of the parasympathetic nervous system. Quantitative evaluation of sympathetic and parasympathetic activity is available (Fig. 3).

(4) Respiration (RSP)

**Figure 4:**
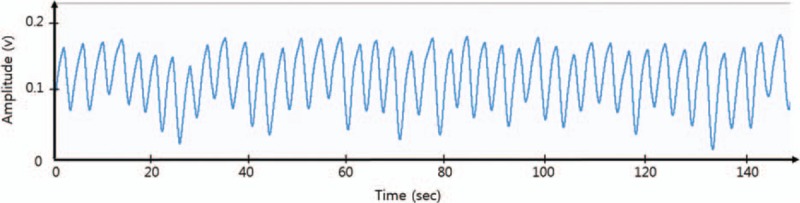
Respiration waves as measured by thermistor according to measurement time.

A fast-response surface temperature thermistor will be attached to the participants’ philtrum region to measure the temperature changes as they inspire and exhale through their nose. The respiration rates will be measured using the flows of the temperature change signals (Fig. 4).

(5) Mean area under photoplethysmogram curve (MAUCppg)

**Figure 5:**
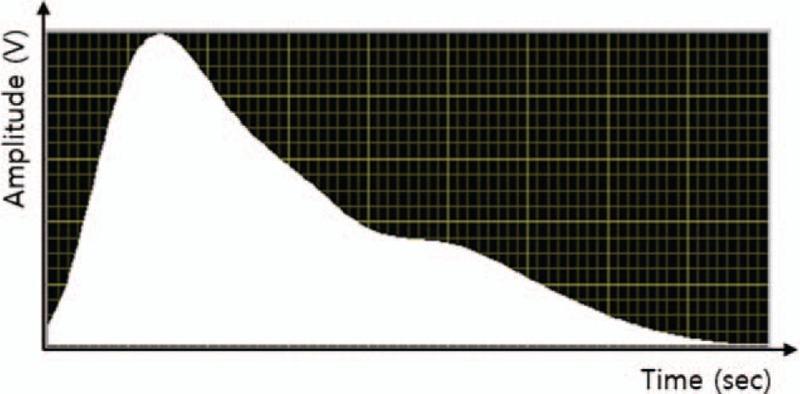
Pulse PPG waveform measured at patient's fingertip as a function of time.

The MAUCppg measuring method estimates the state of heartbeat activities using the optical characteristics of in vivo tissues to capture the changes in blood flow rates and blood vessel volumes caused by the dilatation and contraction of the heart. The mean PPG waveforms will be measured from the starting point to the end point, and the area below the mean waveform curve will be drawn (Fig. 5).

(6) Brachial ankle pulse wave velocity (BaPWV).

The brachial-ankle pulse wave velocity (baPWV) is a unique measure of the systemic arterial stiffness. It is assessed through brachial and tibial arterial wave analyses.^[10]^

(7) Ankle-brachial index (ABI)

The ankle-brachial pressure index (ABPI)-or ankle-brachial index (ABI)-is the ratio of the blood pressure at the ankle to the blood pressure in the upper arm (brachium). Lower blood pressure in the leg than in the arm is an indication of blocked arteries due to peripheral artery disease (PAD). The ABPI is calculated by dividing the systolic blood pressure at the ankle by the systolic blood pressure in the arm:

ABI = SysBPLeg/SysBPArm,

where SysBPLeg indicates the systolic blood pressure in the leg and SysBPArm indicates the systolic blood pressure in the arm.

(8) Surface temperature on hand palms

To investigate the changes in stroke patients’ temperature over the course of the treatment, the temperature will be measured at the center of both palms 3 times. The mean temperature will be used in the analysis.

(9) Body composition analysis

To investigate the correlation between the body composition measurements and the changes in the radial pulse over the course of stroke treatment, the soft lean mass (SLM), total body water (TBW), and body fat mass (BFM) will be measured using Inbody S-10 (InBody, South Korea).

(10) Assessment of walking ability

Variables including the cadence, speed, stride length, and symmetry index will be measured using the G-Walk (BTS S.P.A, Italia, Seoul receiver 12–191) to assess the improvement in the walking ability over the course of the stroke treatment.

(11) Plasma inflammatory index

The plasma high-sensitivity C-reactive protein (hs-CRP) level will be measured.

(12) Thrombosis-related indices

The plasma levels of factors associated with thrombosis/hemostasis (the von Willebrand, D-dimer, and fibrinogen factors) will be measured.

(13) Plasma viscosity measurement

The following variables will be measured to assess the plasma viscosity: the systolic relative viscosity (SRV) – that is, the relative viscosity at which the shear rate (1/s) is 300-and the diastolic relative viscosity (DRV) – that is, the relative viscosity at which the shear rate (1/s) is 5.

(14) Manual muscle testing (MMT)

MMT is performed as a screening test and to identify the relationship with the radial artery pulse wave profile. It provides a baseline for therapy by assessing the potential muscle strength through the measurement of the resistance against gravity and against opposing forces in each muscle. It is used to explore the influence of muscle weakening on the activities of daily living and to select activities that can be performed within the patient's capacity, thus allowing to assess the treatment efficacy.

(15) Korean Version of National Institutes of Health Stroke Scale (K-NIHSS)

The Korean Version of National Institutes of Health Stroke Scale (K-NIHSS) is the most widely used tool for the absolute measurement of post-stroke disability. It comprises 14 categories that assess the cognitive level, gaze, field of vision, facial palsy, limb power, loss of mobility, sensation, language, dysarthria, neglect, and distal upper limb movement.

(16) Beck depression inventory (BDI)

The Beck depression inventory (BDI) is a self-reporting tool comprising 21 categories-including emotional, cognitive, causal, and physiological symptoms-that is used to assess depression in patients. Each of the 21 questions is scored between 0 and 3, for a total between 0 and 63. A score of 0–9 is considered normal, while 10–15 indicates mild depression, 16–23 indicates moderate depression, and 24–63 indicates severe depression.

(17) Blood stasis syndrome questionnaire (BSSQ)

The blood stasis syndrome questionnaire (BSSQ) was developed and validated by the Korea Institute of Oriental Medicine (KIOM) by integrating the Korean, Chinese, and Japanese blood stasis syndrome questionnaire (BSS) criteria in a community-based, multi-center trial.

### 2.8 Statistical analysis

The software package R (version 3.0.1, “R & R” of the Statistics Department of the University of Auckland, Auckland, New Zealand) or STATA version 13.1 (StataCorp, College Station, TX, USA) will be used for the statistical analyses. To ensure sensitivity analyses, analyses of the primary and secondary outcomes will be conducted on the full analysis set (FAS) and per protocol set (PP). The last observation carried forward (LOCF) method will be used to impute the missing data.

Baseline characteristics will be presented using the t-test or Wilcoxon rank-sum test for continuous outcomes, and the chi-square test or Fisher's exact test for categorical outcomes. The differences in the intergroup comparisons of the means will be processed with the least squared mean (LSM) and the standard deviation, and the intragroup comparisons of the mean values will be analyzed with a repeated-measures ANOVA (RMANOVA) or a linear mixed-effects model analysis (LME). *P* < 0.05 will be considered statistically significant. The time point difference in contrast with the baseline will be examined with Dunnett's test.

### 2.9 Data handling

The investigators will enter the information required by the protocol into case report forms (CRFs). Non-obvious errors or omissions will be entered into data query forms that will be returned to the investigational site for resolution. The data from all of the centers will be gathered and summarized into baseline demographic characteristic observations. The participant information will be treated confidentially. The final data set will be accessible by the principal investigator.

### 2.10 Adverse events

The investigators will record adverse events and unexpected responses. Adverse events will be reported by the participants and will be evaluated by the investigators as mild, moderate, or severe according to the World Health Organization Draft Guidelines for Adverse Event Reporting and to Spilker's criteria. When severe adverse event happens, the trial participant will discontinue the trial.

### 2.11 Monitoring

Monitoring will be conducted regularly by a clinical research monitor. At the time of the visit, the monitor will check the original patient record, medical device management record, and data archive (e.g., study file).

## 3 Discussion

The outcome measures of this study will be the differences and changes in the radial artery pressure pulse, in the blood vessel properties, and in the physiological data obtained during the treatment of patients with hemiplegia caused by stroke. The changes in the artery pressure pulse, in the blood vessel properties, and in the physiological data will be compared.

These results can provide information about physiological and hemodynamic mechanisms. They will also help to clarify whether the radial artery pressure pulse wave can be used to evaluate the result of stroke treatment objectively. This study's findings will be scientifically relevant, as they will allow us to quantify the degree to which the PWRA is related to the reduction in daily physical activities in stroke patients with hemiplegia, and to analyze the changes over time. To our knowledge, this will be the first study to compare the PWRA on the affected side and on the normal side of stroke patients with hemiplegia. It will also show the relationship between hemiplegia and the PWRA over a follow- up period of 6 months.

Data on the changes in the pulse wave characteristics of the radial artery and on the course of these changes over time in stroke patients with hemiplegia are currently scarce and inconclusive. To achieve external validity and to provide a standardized Korean Medicine diagnosis system for stroke and to benefit evidence-based medicine, further studies need to assess these factors quantitatively and qualitatively. The present study hopes to contribute evidence to help clarify some of these issues. Its results will be available in February 2019.

## Acknowledgments

This study was supported by a grant from the Clinical Trial Center of Wonkwang University Gwangju Hospital funded by the Ministry of Health & Welfare through the Korea Health Industry Development Institute (KHIDI) (HI14C0665) and the “Development of pulse analysis system for personalized medicine by converging hemodynamics and pulse diagnostics” project (K17021) funded by the Korea Institute of Oriental Medicine.

## Author contributions

**Conceptualization:** J.U. Kim, S. Lee.

**Funding acquisition:** J.U. Kim, S. Lee.

**Investigation:** J-H. Bae.

**Methodology:** B. Ku.

**Software:** B. Ku.

**Supervision:** J.U. Kim, S. Lee, S. Yeom.

**Writing – original draft:** J.K. Kim, J-Y. Shin, J.U. Kim, S. Lee.

**Writing – review & editing:** J.U. Kim, J-H. Bae, S. Lee, S. Yeom.
